# Physical Activity in the School Setting: Cognitive Performance Is Not Affected by Three Different Types of Acute Exercise

**DOI:** 10.3389/fpsyg.2016.00723

**Published:** 2016-05-17

**Authors:** Vera van den Berg, Emi Saliasi, Renate H. M. de Groot, Jelle Jolles, Mai J. M. Chinapaw, Amika S. Singh

**Affiliations:** ^1^Department of Public and Occupational Health and the EMGO Institute for Health and Care Research, VU University Medical CenterAmsterdam, Netherlands; ^2^Welten Institute – Research Centre for Learning, Teaching and Technology, Open University of the NetherlandsHeerlen, Netherlands; ^3^Department of Complex Genetics, School for Nutrition, Toxicology and Metabolism/Faculty of Health, Medicine and Life Sciences, Maastricht UniversityMaastricht, Netherlands; ^4^Centre for Brain and Learning, Faculty of Psychology and Education, LEARN! Institute, Vrije Universiteit AmsterdamAmsterdam, Netherlands

**Keywords:** acute exercise, exercise type, cognition, selective attention, information processing speed, adolescents, school setting

## Abstract

Recent studies indicate that a single bout of physical exercise can have immediate positive effects on cognitive performance of children and adolescents. However, the type of exercise that affects cognitive performance the most in young adolescents is not fully understood. Therefore, this controlled study examined the acute effects of three types of 12-min classroom-based exercise sessions on information processing speed and selective attention. The three conditions consisted of aerobic, coordination, and strength exercises, respectively. In particular, this study focused on the feasibility and efficiency of introducing short bouts of exercise in the classroom. One hundred and ninety five students (5th and 6th grade; 10–13 years old) participated in a double baseline within-subjects design, with students acting as their own control. Exercise type was randomly assigned to each class and acted as between-subject factor. Before and immediately after both the control and the exercise session, students performed two cognitive tests that measured information processing speed (Letter Digit Substitution Test) and selective attention (d2 Test of Attention). The results revealed that exercising at low to moderate intensity does not have an effect on the cognitive parameters tested in young adolescents. Furthermore, there were no differential effects of exercise type. The results of this study are discussed in terms of the caution which should be taken when conducting exercise sessions in a classroom setting aimed at improving cognitive performance.

## Introduction

Schools and teachers experience increased pressure to improve cognitive performance and scholastic achievement of their students (Wilkins et al., 2003; McMullen et al., 2014). This pressure is caused by the high demands that governments place on students’ performance in language and mathematic subjects, which are used for the evaluation and funding of schools (Center for Education Policy, 2007; Dutch Inspectorate of Education, 2015). Consequently, the time allocated to academic subjects can result in reduced time for physical education and physical activity (PA) in the school curriculum (Wilkins et al., 2003; Centers for Disease Control and Prevention, 2010). Recent studies, however, showed that in addition to the well-known physical and mental health benefits (Janssen and LeBlanc, 2010; Biddle and Asare, 2011), regular participation in PA seems to benefit cognitive functioning and scholastic achievement (see for a review Howie and Pate, 2012; Singh et al., 2012; Khan and Hillman, 2014).

In addition to the chronic effects of PA on cognitive performance, a single bout of physical exercise seems to have positive effects on performance in several cognitive tasks (see for a review Tomporowski et al., 2011; Chang et al., 2012). For example, information processing speed (e.g., Ellemberg and St-Louis-Deschênes, 2010; Cooper et al., 2012) and selective attention (e.g., Tine and Butler, 2012; Janssen et al., 2014a) have been shown to improve immediately after a single exercise session. Information processing speed refers to the efficiency of executing cognitive tasks and is associated with cognitive performance (Kail and Ferrer, 2007). Selective attention is needed to ‘select and focus on particular input for further processing, while simultaneously suppressing irrelevant or distracting information’ (Stevens and Bavelier, 2012, p. 30). The above-mentioned cognitive functions play an important role in classroom behavior and learning processes and thereby contribute to scholastic achievement (Rohde and Thompson, 2007; Stevens and Bavelier, 2012). Therefore, conducting exercise sessions during the school day may yield immediate positive effects on learning efficiency in the classroom.

Several neurobiological mechanisms have been hypothesized to explain the acute effects of exercise on cognitive functioning: (1) increased blood flow to the brain thereby elevating oxygen uptake (Ogoh and Ainslie, 2009); (2) increases in neurotrophic factors, such as brain derived neurotrophic factor, growth hormone, and insulin-like growth factor-1 (Gregory et al., 2013; Piepmeier and Etnier, 2014); (3) increases in brain neurotransmitters, such as dopamine, norepinephrine, and serotonin, which results from an exercise-induced increase in arousal that involves the activation of the autonomic nervous system and hypothalamic-pituitary-adrenal axis (McMorris et al., 2009; McMorris and Hale, 2014). According to the arousal theory, the exercise-induced arousal improves cognitive performance in an inverted U-shape fashion (Yerkes and Dodson, 1908).

Although there is emerging evidence of positive effects of acute exercise on cognition, the characteristics of exercise needed to improve cognitive functioning in children and adolescents remain largely unknown (Howie and Pate, 2012; Verburgh et al., 2014). A review of Chang et al. (2012) suggested that a minimum duration of 11 min is needed for obtaining relevant cognitive benefits. However, little is known regarding the *optimal type* of acute exercise that benefits cognition most. While the majority of studies among children and adolescents have focused on aerobic exercise, only few studies (e.g., Budde et al., 2008; Gallotta et al., 2012, 2015) compared regular, repetitive aerobic exercises (e.g., walking, running) with more complex, cognitively demanding exercises (e.g., coordinative exercises). For example, Budde et al. (2008) found improved selective attention in adolescents (age 13–16 years) after both a 12-min aerobic and coordinative exercise session, with highest improvements in the coordination group. Coordinative exercises are believed to improve selective attention by pre-activation of cognitive related neuronal networks (Budde et al., 2008). According to Budde et al. (2008), there is an overlap in functional brain areas supporting motor functions and selective attention, such as the frontal lobes and the cerebellum. Higher motor demands, as in coordinative exercises, are suggested to require higher prefrontal cortex activity, thereby facilitating activation of neuronal networks responsible for attention performance (Budde et al., 2008). In contrast, Gallotta et al. (2012, 2015) found larger improvements in selective attention in 8–11 years old children after aerobic compared to coordinative exercise. Bailey et al. (2014) found no acute effects of 15 min coordination or aerobic exercises on selective attention in young adults.

To our knowledge, no published studies report on the acute effect of strength exercise on cognitive performance in children and adolescents. However, studies in adults have shown positive effects of a single bout of strength exercises on selective attention (Chang and Etnier, 2009; Alves et al., 2012; Chang et al., 2014) and information processing speed (Chang and Etnier, 2009; Chang et al., 2014).

The growing literature on the acute effects of exercise on cognition leads several researchers advocate the implementation of exercise sessions in schools (e.g., Hillman et al., 2009; Drollette et al., 2012). However, most evidence of acute exercise effects on selective attention and information processing speed is derived from studies in laboratory settings (e.g., Hillman et al., 2009; Ellemberg and St-Louis-Deschênes, 2010; Drollette et al., 2012). More studies in a school setting are needed in order to generalize these results into practice (Janssen et al., 2014b). Since there is only a limited number of studies in a school setting that examined exercise related effects on information processing speed and selective attention in young adolescents (e.g., Cooper et al., 2012, 2013; Tine and Butler, 2012; Janssen et al., 2014a), this study will focus on 10–13 years old.

Taken together, there is limited and inconclusive evidence regarding the differential effects of exercise types on cognitive functioning in children. In addition, there is a need for more studies in young adolescents conducted in a school setting. Therefore, the aims of this study were to examine (1) the acute effects of 12-min classroom-based exercise sessions on cognitive tasks that measure information processing speed and selective attention in young adolescents aged 10–13 years; and (2) the moderating effects of type of exercise, i.e., aerobic, coordination, or strength.

Given the inconsistencies in studies comparing the differential effects of exercise type on cognition, but mainly positive effects of each individual exercise type, we expect to find similar acute effects of different types of exercise on measures of information processing speed and selective attention in 10–13 years old.

## Materials and Methods

### Recruitment and Participants

A convenience sample of three regular public primary schools in The Netherlands participated in this study. In order to test classroom-based exercise sessions, all children enrolled in the 5th and/or 6th grade were eligible to participate. In total, eight classes participated, three grades 5 and five grades 6 divided over the three schools. The number of classes in each school varied from 1 to 5. All classes were invited and agreed to participate after receiving information on the nature and scope of the study. The principals of the three schools signed an informed consent for participation of all students in their grades 5 and 6. Upon principal agreement children and parents/legal guardians received information letters about the study and could withdraw their child from participation by signing and returning an objection form. Participation in this study was voluntary; children received a small incentive for their participation. The Ethics Committee of the Faculty of Human Movement Science of the VU University Amsterdam, The Netherlands concluded that our study does not fall within the scope of Medical Research Involving Human Subjects Act. They approved the study protocol and agreed with active informed consent by the school principal with parents having the option of opting their child out of the study.

### Study Design and Procedure

The current study had a double baseline, mixed within- and between-subjects design. All students participated in one familiarization day and two experimental days: one exercise day and one control day, thereby acting as their own control. The exercise as well as the control day consisted of a pre- and post-test, by which we controlled for intra-individual differences in cognitive test performance across days. In order to minimize potential learning effects due to test repetition, we included a familiarization day and counterbalanced the order of the experimental days. During the familiarization day, students were acquainted with the test procedures and practiced the tests to make sure they clearly understood them. Furthermore, we controlled for potential learning effects by randomizing and counterbalancing the order of exercise day and control day across classes. By means of the counterbalanced design we were able to estimate effects of exercise more accurately. Three classes started with the control day in week 1 and exercise day in week 2, while five other classes started with the exercise day in week 1 and control day in week 2. Type of exercise acted as between-group factor. Each class was randomly assigned to one of three exercise types (see Exercise Session). Three classes were assigned to the aerobic condition, three classes to the coordination condition and two classes to the strength condition. The experiment took place in the classroom and was procedurally the same for each class.

**FIGURE 1:**
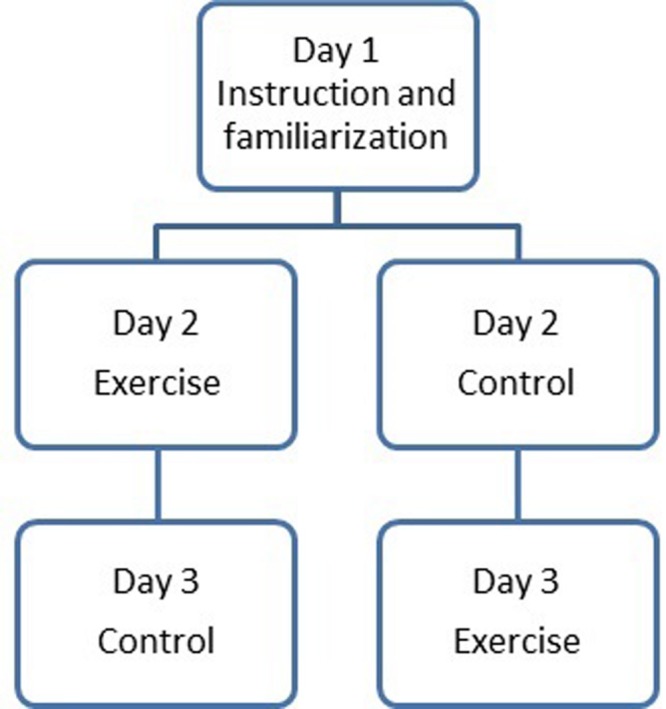
**Overview study design**.

All classes were visited three times (see **Figure 1**). During the first visit the experimental protocol was explained and students were familiarized with the measurement procedures. The cognitive tests were introduced and practiced by the students. Height and weight were measured in a separate room outside the classroom. The subsequent visits consisted of the two experimental days, which were scheduled one week apart, at the same day of the week from 08:30 to 10:00 a.m.

**FIGURE 2:**
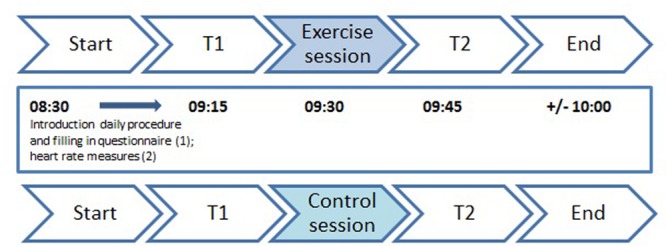
**Experimental protocol during exercise day (above) and control day (below)**.

Both experimental days followed a standardized routine, completed in the classroom setting (see **Figure 2**); (1) we informed the students about the daily procedure and asked them to fill in a short questionnaire about their bedtime, breakfast and transport to school; (2) students were fitted with a heart rate (HR) monitor and their resting HR was measured (exercise day) or students learned how they could measure their own HR at the wrist or neck (control day); (3) we asked to complete the pre-cognitive tests (T1), followed by the exercise or control session, each lasting 12 min; (4) immediately after ending of the session, administration of the post-cognitive tests (T2) took place.

### Bedtime, Breakfast, and Transport to School

In order to keep the circumstances in both experimental days similar, we asked students and their parents/legal guardians to try to keep bedtime, breakfast and transport to school approximately the same before each experimental day.

### Exercise Sessions

Together with two physical education teachers we developed three different types of exercise sessions. Each session lasted 12 min. The first 1½ min (warming-up) and last 30 s (cooling-down) were equal for all exercise types. The core of the sessions consisted either of 10 min aerobic, coordination, or strength exercises. Exercises were intended to be of moderate intensity and easy to perform in the classroom behind students’ desks.

The aerobic session consisted of various well-known, easy and repetitive movements. The coordination session consisted of more complex movements that stressed coordinative skills, including bilateral movements and movements in which the body mid line was crossed. The strength session consisted of dynamic and static body-weight exercises, adjusted to the age of the students (see **Table 1**).

**Table 1:** Examples of exercises within each exercise type.

Aerobic exercises	Coordination exercises	Strength exercises
-Walking, marching, dribbling, or jogging in place. -Jumping in place (pretending to jump rope, pretending to throw a ball in the basket). -Pretend to: swim freestyle, throw and kick a ball, riding on a hand-bike and ice skating.	-Dance steps with different directions and speed. -Walking in place and simultaneously touching the opposite ankle in front or behind the body. -Head, shoulders, knees and toes with different order, and touching opposite body side. -Clapping hands under the legs, while standing straight, and while rotating legs to the side.	-Squats (‘bend your knees until you are almost sitting’) -Abdominal exercises while sitting on chair (e.g., knee lifting, extending legs) and while standing (bending upper body aside; move knee and elbow toward each other). -Heel raise, shoulder side raise, and arm circles. -Dynamic and static bicep curls (‘make your muscles as big as you can!’).

In order to standardize the exercise sessions, a movie of each session was recorded beforehand. The movie was shown in the classroom and students were asked to follow and imitate the instructor in the movie. One researcher and three research assistants were present during the exercise sessions, to motivate and guide the students.

### Control Session

In the control condition, students were seated during 12 min and listened to an educational lesson about exercise and movement. This lesson was always taught by the same research assistant and interaction with students was kept to a minimum. Students were allowed to ask a few questions during the last minute of the lesson.

### Measures

#### Participant Information

We assessed age and sex by self-report. Body weight and height were measured according to standardized protocols with the students wearing regular clothes without shoes. Body Mass Index (BMI) was calculated [weight (kg)/height(m)^2^].

#### Heart Rate

During the exercise session, students wore a HR monitor (Polar H7 Bluetooth) that was connected to the Polar Team App (Polar Electro Oy, Finland). Resting HR (HR rest) was measured after students sat still for 5 min. HR during the exercise sessions was measured and stored in the Polar Team App. Mean HR during the exercise sessions was calculated (HR exercise). The formula ‘220 – age’ was used to estimate maximum HR (HR max; American College of Sports Medicine [ACSM], 2013).

Intensity of the exercise sessions was calculated as percentage of HR max: (HR exercise/HR max)^∗^100. Percentage of time spent in moderate to vigorous intensity zone (64–94% HR max; American College of Sports Medicine [ACSM], 2013) was calculated for the 10 min aerobic, coordination, and strength exercises.

#### Cognitive Measures

Cognitive performance was measured with two neuropsychological tests, the d2 Test of Attention (Brickenkamp and Oosterveld, 2012) and the Letter Digit Substitution Test (LDST) (van der Elst et al., 2006; Van der Elst et al., 2012). Both paper-and-pencil tests were group-administered. Before each test, students were provided with standardized verbal and written instructions. During testing, the test leader gave instructions and kept track of time, while three research assistants each observed a group of students and took notes of disturbances (e.g., noise outside the classroom), deviant behavior (e.g., students ignoring test instructions), and practical problems (e.g., students having an empty pen).

#### Letter Digit Substitution Test (LDST)

The LDST is a substitution test that measures general information processing speed (Van der Elst et al., 2012). It is an adaptation of earlier substitution tests, such as the Symbol Digit Modalities Test (Smith, 1982) and the Digit Symbol Substitution Test (Wechsler, 1981). The LDST requires students to match letter-number pairs according to a key, which is presented on top of the sheet. The key contains nine boxes with letters and associated numbers, between 1 and 9. Underneath the key, boxes of letters are shown with empty boxes below. Students were instructed to fill in the empty boxes with the appropriate numbers as fast and accurately as possible within 90 s. The number of correct substitutions made in 90 s was used as dependent variable. The test–retest reliability of the LDST has proven to be high in a large sample of adults (*r* > 0.85) (Van der Elst et al., 2008). Furthermore, studies have shown that the LDST is sensitive to age, sex, and education level in children and adolescents aged 8–15 years (Van der Elst et al., 2012; Dekker et al., 2013). Four different versions of the LDST were administered during the four test moments. The order of test versions was equal for all students.

#### d2 Test of Attention

The d2 Test of Attention is a cancelation task that measures selective attention (Brickenkamp and Oosterveld, 2012). The test consists of one page with 14 lines, each consisting of 47 letters ‘d’ and ‘p’. Above and/or below each letter are 1–4 dashes displayed, either individually or in pairs. Students were instructed to mark as much letters ‘d’ with a total of two dashes (‘d2’) within each line, while ignoring all other characters. A ‘d2’ has either two dashes above, two dashes below or one dash above and one dash below the ‘d’. Students were instructed to work from left to right, with a time limit of 20 s per line. After 20 s, the test leader gave a signal to continue with the next line. The total test lasted 4 min and 40 s.

Three different parameters can be calculated after completion of the d2 test. First, the total number of items processed, which is a measure of working speed. Second, the number of all errors relative to the total number of items processed, a measure of precision and thoroughness. Third, the number of correctly marked d2 characters minus the number of incorrectly marked characters, which is a measure of attention span and concentration ability. In this study, we used the latter as dependent variable. This value is, opposed to the other values, resistant to falsifications and therefore an objective measure of selective attention (Brickenkamp and Oosterveld, 2012). The test–retest reliability of the d2 test has been proven to be moderate to high in a population of 144 Dutch children, aged 10–13 years (*r* = 0.79–0.83) (Brickenkamp and Oosterveld, 2012).

#### Evaluation of the Exercise Sessions

To evaluate students’ experience regarding the difficulty and enjoyment of the three exercise sessions, students were asked two questions: (1) How much did you like the exercise session? (‘fun’); (2) How difficult was the exercise session? (‘difficulty’). Answers were given on a five-point Likert scale, ranging from 1 ‘not at all’ to 5 ‘very much.’

### Statistical Analysis

All statistical analyses were performed in SPSS version 20.0. One-way ANOVAs and Chi-square tests were conducted to compare student characteristics and HR data between the exercise types. Cognitive outcomes were analyzed using 2 × 2 × 3 mixed ANOVAs with time (T1,T2) and condition (control, exercise) as within-subject variables and exercise type (aerobic, coordination, strength) as between-subject variable. In order to control for session order (control-exercise versus exercise-control), this variable was included as additional between-factor in all analyses. Analyses were conducted separately for the LDST and d2 test. *Post hoc* comparisons (Bonferroni adjusted) were conducted in case of significant findings. Level of significance was set at α < 0.05.

## Results

One hundred and ninety five students from eight classes of three primary schools in the Netherlands participated. Students were in 5th and 6th grade and their age ranged from 10 to 13 years. Two students’ parents returned the objection form and their children were therefore excluded from the study.

Eleven students were excluded from analyses due to absence at one or both experimental days. An additional number of four (LDST) and five (d2 test) students were excluded based on invalid test scores due to missing a part of the test (one student came in later, two students had an empty pen during the test) or ignoring test rules (students who did not follow the start/stop instructions). The final dataset consisted of 180 students in the LDST analysis and 179 students in the d2 test analysis.

### Student Characteristics

Demographics of the total group and for each exercise type group are shown in **Table 2**. *Post hoc* multiple comparisons showed higher mean age and height for the strength group. HR exercise, %HR max and percentage in moderate-to-vigorous intensity zone were significantly higher during the aerobic exercise session. Baseline test scores for the LDST and d2 test did not differ between exercise types.

**Table 2:** Characteristics of the total sample and three exercise types (means, standard deviations (SD), percentages).

	Total (*n* = 184)	Aerobic (*n* = 66)	Coordination (*n* = 71)	Strength (*n* = 47)
Age (years)	11.7 (0.7)	11.6 (0.7)	11.7 (0.8)	12.1 (0.5)*ac
Sex (boy/girl; %)	54/46	53/47	56/44	51/49
Height (cm)	154.6 (7.5)	152.8 (7.8)	154.3 (6.9)	157.4 (7.2)*a
Weight (kg)	44.1 (8.6)	43.1 (7.7)	43.6 (8.5)	46.3 (9.6)
BMI	18.4 (2.4)	18.5 (2.2)	18.2 (2.6)	18.5 (2.5)
HR exercise (beats/min)	120.1 (12.5)	127.0 (12.6)*cs	114.1 (10.6)	119.7 (9.9)*ac
%HR max	57.7 (6.0)	60.9 (6.0)*cs	54.8 (5.1)	57.6 (4.8)*ac
Moderate-to-vigorous zone (% of core 10 min)	24.4 (24.8)	39.5 (27.0)*cs	14.1 (17.3)	18.8 (20.9)
Familiarization day LDST	47.2 (8.9)	45.2 (8.7)	46.8 (8.5)	50.6 (8.8)
Baseline LDST	49.2 (8.7)	47.7 (8.7)	49.5 (8.8)	51.1 (8.5)
Familiarization day d2	142.1 (22.5)	141.3 (24.2)	139.8 (20.4)	146.5 (22.8)
Baseline d2	166.5 (26.0)	163.6 (26.3)	165.1 (25.2)	172.7 (26.2)
Perceived fun of exercise	3.9 (0.9)	3.6 (0.9)	4.0 (0.9)	3.9 (0.7)
Perceived difficulty of exercise	1.9 (1.0)	1.6 (0.8)	2.2 (1.1)	1.8 (0.9)

*Baseline LDST and d2 scores are pre-test scores at the first experimental day. ^∗^a, significantly different from aerobic group (*p* < 0.05); ^∗^ac, significantly different from aerobic and coordination group (*p* < 0.05 and *p* < 0.001); ^∗^cs, significantly different from coordination and strength group (*p* < 0.001 and *p* < 0.05).*

### Cognitive Performance

Due to differences in age for exercise type, this variable was added as covariate in all analyses. After controlling for age and session order, there were no significant acute effects of exercise on information processing speed [*F*(1,174) = 0.71, *p* = 0.40, $\eta_{p}^{2}$ = 0.00] and selective attention [*F*(1,172) = 0.91, *p* = 0.34, $\eta_{p}^{2}$ = 0.01]. Likewise, type of exercise did not moderate effects on information processing speed [*F*(1,174) = 1.75, *p* = 0.18, $\eta_{p}^{2}$ = 0.02] and selective attention [*F*(1,172) = 0.60, *p* = 0.55, $\eta_{p}^{2}$ = 0.01]. Pre- and post-test scores showed similar patterns in the exercise and control day, and did not differ between exercise types (**Table 3**).

**Table 3:** Letter Digit Substitution Test (LDST) and d2-test scores at T1 and T2 during the control and exercise day for the total group and three exercise types, controlled for session order and age (means and standard errors of the means).

Letter Digit Substitution Test								
	Total (*n* = 180)		Aerobic (*n* = 65)		Coordination (*n* = 71)		Strength (*n* = 44)	
	Control	Exercise	Control	Exercise	Control	Exercise	Control	Exercise
T1	51.7 (0.8)	51.7 (0.7)	49.5 (1.3)	49.8 (1.2)	51.4 (1.2)	50.5 (1.2)	54.3 (1.5)	54.7 (1.5)
T2	51.8 (0.6)	51.9 (0.7)	51.1 (1.1)	50.4 (1.1)	49.4 (1.0)	50.4 (1.1)	54.9 (1.3)	54.9 (1.3)

**d2-Test of Attention**								

	**Total (*n* = 179)**		**Aerobic (*n* = 63)**		**Coordination (*n* = 69)**		**Strength (*n* = 47)**	
	**Control**	**Exercise**	**Control**	**Exercise**	**Control**	**Exercise**	**Control**	**Exercise**

T1	181.5 (2.5)	182.9 (2.3)	175.6 (4.2)	178.4 (3.9)	179.5 (4.0)	181.5 (3.7)	189.4 (4.8)	188.7 (4.4)
T2	200.4 (2.9)	203.2 (2.8)	194.9 (5.0)	199.1 (4.8)	199.2 (4.8)	200.9 (4.5)	207.1 (5.7)	209.5 (5.4)

For both cognitive tasks, there was a significant interaction of session order. Independent of exercise or control day, information processing speed scores increased during day 1 and decreased during day 2. Selective attention scores improved significantly more during day 1 than during day 2.

## Discussion

The purpose of the current study was to examine the acute effects of single classroom-based exercise sessions on information processing speed and selective attention, and differences in effects between three different exercise types (i.e., aerobic, coordination, and strength).

### Acute Effects of Exercise on Cognition

There was no support for the notion that acute physical exercise improves cognitive performance, as there was no significant overall acute effect of 12 min exercise on a selective attention and information processing test in 10–13 years old children. There was neither a differential effect of one of the exercise types on students’ cognitive performance.

The current results are partly in line with Hill et al. (2010), who found no significant main effect of 10–15 min classroom-based exercise on a digit-symbol coding task. However, a significant interaction in their analysis indicated that there was an acute effect of exercise for children who followed the exercise session in the second week of the counterbalanced experiment. In line with our current results, one laboratory study with a duration of 20 min (Stroth et al., 2009), and two school-based studies with exercises of 5 min (Kubesch et al., 2009) and 45 min (Pirrie and Lodewyk, 2012) neither found an acute effect of exercise on children’s attention performance.

In contrast to our results, other published studies conducted in a school setting reported acute effects of 10–15 min aerobic exercise on information processing speed and selective attention in comparison to a control condition (e.g., Cooper et al., 2012, 2013; Tine and Butler, 2012; Niemann et al., 2013; Janssen et al., 2014a). The inconsistencies in results of the aerobic exercise in the current and other studies may be due to differences in exercise intensity. The aerobic exercise session in the current study turned out to have low to moderate intensity for the average student in the classroom (mean: 61% HR max; 39.5% of time spent in moderate to vigorous intensity zone). In contrast, the positive result studies implemented aerobic exercises with moderate to vigorous intensity (mean HR: 172 and 169 beats/min in 11–13 years old in Cooper et al., 2012, 2013; 70–85% HR max in Tine and Butler, 2012 and Niemann et al., 2013; 2000–2999 and >3000 counts/minute, indicating moderate and vigorous intensity in Janssen et al., 2014a). According to the arousal theory, highest cognitive improvements are expected to occur at moderate intensity levels (McMorris and Hale, 2012). In this respect, the time spent in moderate to vigorous intensity within the aerobic exercise in the current study might have been insufficient to cause significant improvements in information processing speed and selective attention. This was supported by findings of a recent meta-analysis indicating effect sizes close to zero after a single bout of low intensity exercise in adults (McMorris and Hale, 2012). The necessity to exercise sufficient time at moderate intensity could have important implications for further research and implementation of exercise sessions in schools, since monitoring exercise intensity may not be feasible in a real-life school setting.

Another possible explanation for the inconsistency in results includes the timing of the cognitive testing. In the current study, cognitive tests were conducted before and immediately after the exercise session. In contrast, in the studies of Niemann et al. (2013) and Janssen et al. (2014a) students attended academic classes before start of the experiment. In the studies of Cooper et al. (2012, 2013), the post-test was conducted 10, 45, and 60 min after ending of the exercise session. The influence of the timing of cognitive testing on cognitive outcomes in children is still unclear and needs further investigation (Hillman et al., 2011).

Further, there was no differential effects of exercise type on performance on the two cognitive tests. This is in line with a previous study of Bailey et al. (2014), who neither found acute, nor differential effects of 15 min aerobic and coordinative exercise on the same selective attention test (d2 test). However, this study differed with respect to age of the participants (young adults). Our findings are in contrast to an earlier study in adolescents, reporting higher improvements in selective attention after coordinative versus aerobic exercise (Budde et al., 2008). However, it is important to note that this study included no control group and was conducted in a selective population of elite athletes. The generalizability of the results to regular students is therefore questionable. Moreover, neither our, nor the studies of Gallotta et al. (2012, 2015) and Bailey et al. (2014) were able to replicate the findings of Budde et al. (2008). This might suggest that the role of exercise type may not be as prominent as suggested by Budde et al. (2008).

The current results are also inconsistent with the results of Gallotta et al. (2012, 2015), who reported improved attention after both aerobic and coordinative exercise, with largest improvements after the aerobic exercise. Differences in results may be due to differences in age (8–11 years), duration (30 min within a physical education class of 50 min) and intensity of the exercises (mean HR: 146, moderate to vigorous intensity). However, it is worth mentioning that the improvement in attention was equal (Gallotta et al., 2012) and even larger (Gallotta et al., 2015) following a sedentary academic lesson as compared to the exercise conditions. Comparisons with studies on the acute effect of strength exercises on cognition in children and adolescents could not be made due to absence of studies in this age group.

The lack of effects in the current study may raise questions with regard to the generalizability of the results from lab-based studies into the classroom. The earlier, positive result studies in a school-setting conducted short exercise sessions outside the classroom (Budde et al., 2008; Cooper et al., 2012, 2013; Tine and Butler, 2012; Niemann et al., 2013). In contrast, the current and other studies that reported no acute effects of exercise on attention and information processing speed (5 min movement break in Kubesch et al., 2009; 10–15 min exercise within week 1 in Hill et al., 2010), implemented exercise sessions within the classroom. Although Kubesch et al. (2009) and Hill et al. (2010) did not report the intensity of their exercise sessions, the low to moderate intensity of the exercise sessions in our study indicate that it might be difficult to reach sufficient intensity when exercising in a classroom setting. Therefore, we recommend future studies to monitor and report on exercise intensity to gain more insight in the acute effects of exercise on cognitive performance.

### Strengths and Limitations

Strengths of the current study include the double baseline design with repeated measures. By means of a pre- and post-test design, we were able to control for intra-individual differences across measurement days. Another strength includes the standardized execution of the experiment in a classroom setting, by which we contribute to the generalization of outcomes from previous laboratory studies into the school setting.

Limitations include the measurement of exercise intensity in the strength group. Due to the nature of the exercises (i.e., body-weight exercises), intensity could not be determined by percentage of one-repetition maximum. However, the absence of weight load suggests low to moderate intensity strength exercises. The HR measured at rest turned out to be an inappropriate measure. Part of the children seemed not able to relax completely, possibly due to unfamiliarity with the experimental setting. For this reason, exercise intensity was determined based on an estimation of children’s HR max, without controlling for individual differences in HR rest.

Despite the use of a double baseline, selective attention scores (d2 test) improved significantly more from pre- to post-test during the first experimental day compared to the second day, regardless of exercise or control session. The test–retest reliability of the d2 test has been found moderate to high over a 1 year period (Brickenkamp and Oosterveld, 2012), but it is questionable if this holds for short test-retest intervals and multiple test repetitions, as used in the current study. As these improvements seem to indicate a general learning effect, we recommend future studies to include a control condition other than exercise to be able to discriminate effects of exercise from a general learning effect.

## Conclusion

In summary, the current results suggest that sessions of 12-min classroom-based exercise at low to moderate intensity have no acute effects on information processing speed and selective attention compared to a sedentary control condition in young adolescents. Likewise, no significant differential effects of aerobic, coordination or strength exercises were found. The execution of exercise sessions in the classroom seems feasible, but it might be difficult to reach sufficient intensity in order to gain cognitive benefits.

## Author Contributions

Conception and design of the study: VB, ES, RG, JJ, MC, and AS. Acquisition of the data: VB and ES. Data analysis and interpretation: VB and ES. Drafting the manuscript: VB. Critical revision of the draft for important intellectual content: ES, RG, JJ, MC, and AS. All authors approved the final version of the manuscript for publication and agree to be accountable for the accuracy and integrity of all aspects of the work.

## Conflict of Interest Statement

The authors declare that the research was conducted in the absence of any commercial or financial relationships that could be construed as a potential conflict of interest.
